# Scaling up HIV treatment -Karnataka, India experience

**DOI:** 10.1186/1742-4690-9-S1-P71

**Published:** 2012-05-25

**Authors:** Salma Fahim, Suresh Shastri, Reynold Washington

**Affiliations:** 1 Karnataka State Aids Prevention Society, Bangalore, India; 2 Karnataka Health Promotion Trust, Bangalore, India

## Background

Karnataka a high HIV prevalence state in south India is home to 10 percent India’s HIV infected. The Government of India sponsored HIV treatment program was initiated on 1st April 2004. A systematic approach to scale up of the ART services is followed in Karnataka where Government is the lead agency to implement the program and all the donors/NGOs compliment the program.

## Methods

A donor supported technical consultant was identified and located within the State AIDS Society. A logistic Management Information System is established to track procurement, distribution and supplies. Human resource recruitment, training and retention at the ART centres are decentralized to the district AIDS prevention and control units (DAPCU). Centralized classroom based training is complemented with field based onsite training and mentorship. A Google group is created for technical updates. DAPCU conducts coordination meetings to geographically distribute and allocate responsibility to all field level workers in HIV prevention and care programs to minimize loss to follow up.

## Results

By Sept 2011, 189,179 persons living with HIV (PLHIV) are registered at ART centres and 64,104 are currently on ART. This was possible thanks to a scale up in the numbers of ART centres from 17 to 44 and CD 4 testing machines from 5 to 32 during the period 2008-2011. The proportion of PLHIV detected at Integrated Counseling and Testing Centres and registered in ART centres rose from 46% to 96% in this period. 122 link ART centres are established to decentralize drug distribution for those stable on ART. Lost to follow up among those on ART reduced from5.5% to 3.5%. However, death rates among those on ART remain high at around 17 %. The commonest cause of death is TB (21%), while unknown remains high (26%).

## Conclusions

Despite a rapid scale up, loss to follow up of those initiated on ART has been significantly reduced and stabilized. This was possible because of coordination between government and civil society partners. The high death rates indicate the need for better integration between HIV and TB programs, strengthening clinical competencies, laboratory diagnostic facilities for opportunistic infections and operations research.

